# Pleural Hemangioma Detected by ^18^ F-PSMA PET/CT in High-Risk Prostate Cancer Staging: A Case Report and Review of the Literature

**DOI:** 10.1055/s-0045-1813679

**Published:** 2025-11-23

**Authors:** André Marcondes Braga Ribeiro, Cíntia Natália Gotardo, Stephania Martins Bezerra, Lizieux Matos Fernandes

**Affiliations:** 1 Department of Nuclear Medicine, A. C. Camargo Cancer Center, São Paulo, Brazil; 2 Department of Anatomic Pathology, A. C. Camargo Cancer Center, São Paulo, Brazil; 3 Department of Radiology, A. C. Camargo Cancer Center, São Paulo, Brazil

**Keywords:** ^18^ F-PSMA PET/CT, case report, pitfall, pleural hemangioma, prostate cancer

## Abstract

Prostate cancer (PCa) staging has advanced significantly with the emergence of prostate-specific membrane antigen (PSMA) positron emission tomography/computed tomography (PET/CT). However, PSMA-labeled radiotracers uptake is not exclusive to PCa, leading to potential pitfalls. We report an unusual case of a 71-year-old man with high-risk PCa undergoing initial staging with PET/CT using ^18^ F-labeled PSMA ( ^18^ F-PSMA), which revealed an unexpected uptake in a pleural nodular lesion. Given the extreme rarity of PCa pleural metastases, an excisional biopsy of the lesion was performed. Histopathological analysis confirmed the diagnosis of a pleural lobular capillary hemangioma, a benign vascular tumor extremely rare in this location. This case represents the first documented instance of PSMA radiopharmaceutical uptake in a pleural hemangioma, expanding the spectrum of known PSMA PET/CT pitfalls. This finding underscores the importance of histopathological confirmation for atypical PSMA PET/CT findings in unusual locations, preventing incorrect staging and avoiding inappropriate therapeutic decisions.

## Introduction

Prostate cancer (PCa) is one of the most prevalent malignant neoplasms in males. 1 According to estimates from the National Cancer Institute for the 2023 to 2025 triennium, an average of 71,730 new cases are expected to be detected annually in Brazil. 2 Globally, the disease burden is equally significant: GLOBOCAN reported approximately 1.4 million new diagnoses in 2022. 1 Although many prostatic tumors have an indolent course, a portion exhibit aggressive behavior, with metastatic potential and a significant impact on mortality. Therefore, accurate staging at the time of diagnosis is fundamental.

In this context, positron emission tomography/computed tomography (PET/CT) using radiopharmaceuticals linked to prostate-specific membrane antigen (PSMA) has revolutionized the management of PCa patients, being a molecular imaging tool with high sensitivity and specificity. It is particularly useful in the initial staging of high-risk patients and in the evaluation of biochemical recurrence. 3 The ability to detect lymph node, visceral, or bone metastatic disease, often hidden by conventional imaging methods, allows for more precise and individualized therapeutic planning. 4 5 However, despite its high diagnostic accuracy, PSMA radiopharmaceutical uptake is not exclusive to prostatic neoplastic tissue. The designation “specific” in the antigen's name can be misleading, as PSMA, a folate hydrolase, is also expressed in various normal tissues. Furthermore, increased uptake of PSMA radiotracers can be observed in a variety of benign conditions and nonprostatic neoplasms, potentially leading to false-positive results or incidental findings that may mimic PCa metastases, termed “pitfalls.” 6 7

In this report, we present an unusual case of a patient with a diagnosis of high-risk PCa undergoing initial staging with PSMA PET/CT, which showed increased PSMA expression not only in the prostate but also in a pleural nodular lesion that was ultimately found to be a pleural capillary hemangioma. Hemangiomas are benign vascular tumors that occur rarely in the pleura. Literature describes few cases of pleural hemangioma, often discovered incidentally or as a cause of pleural effusion. 8 To our knowledge, there are no previous reports in literature of PSMA radiopharmaceutical uptake in a pleural hemangioma, making this finding an unprecedented pitfall for PSMA PET/CT in PCa staging. The objective of this report is to describe this rare case, discuss the possible mechanisms for increased PSMA expression in pleural hemangioma, and reinforce the importance of histopathological confirmation of atypical or unusually located findings on PSMA PET/CT, even in high-risk PCa patients.

## Case Report

A 71-year-old man presented with a prostate-specific antigen (PSA) level of 3.93 ng/mL at a follow-up appointment in June 2024. Due to the significant increase in the PSA value compared with his latest exam (1.7 ng/mL in November 2021), a prostate magnetic resonance imaging (MRI) and a new PSA test were requested. The biparametric prostate MRI showed an area in the left mid posterolateral peripheral zone of the prostate with a high probability of clinically significant neoplasia, PI-RADS 4. The PSA was quantified at 4.8 ng/mL.

**Fig. 1:**
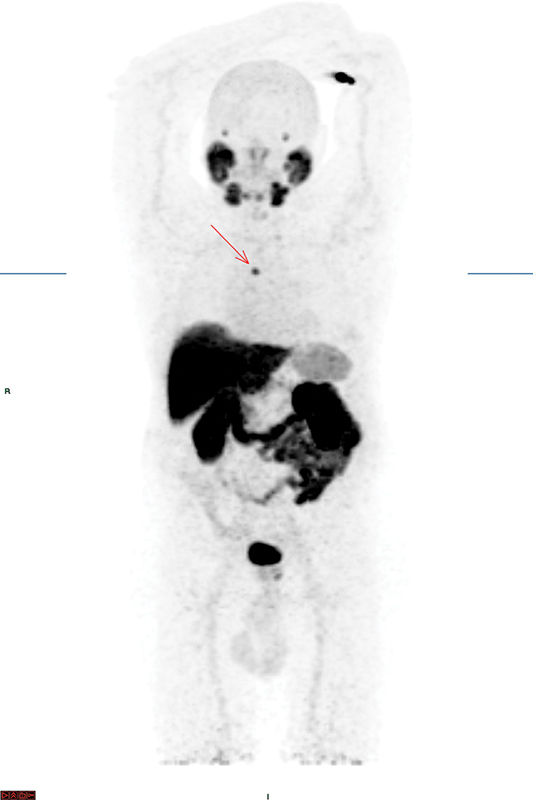
Full-body image in the maximum intensity projection (MIP) showing the abnormal uptake of ^18^ F-labeled prostate-specific membrane antigen ( ^18^ F-PSMA) in the pleural hemangioma (red arrow).

**Fig. 2:**
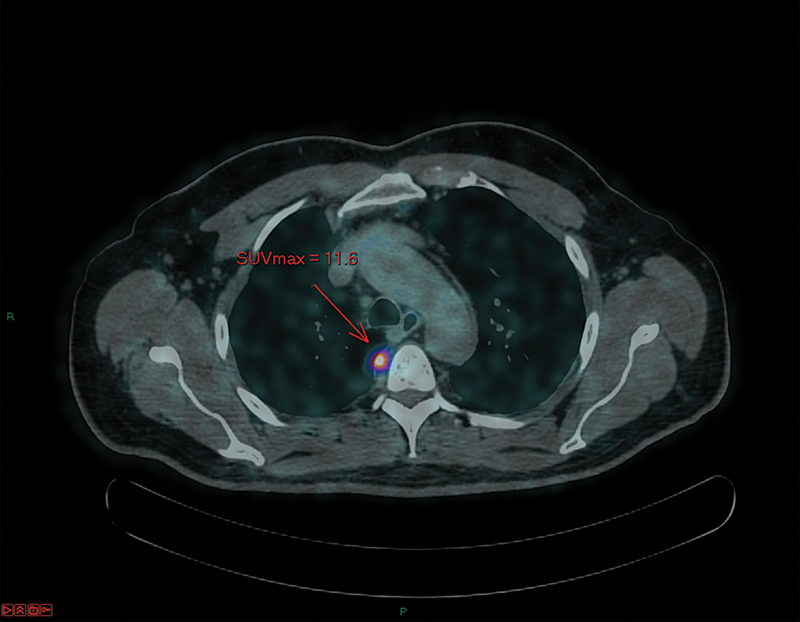
Axial slice from positron emission tomography/computed tomography (PET/CT) fusion demonstrating the uptake of ^18^ F-labeled prostate-specific membrane antigen ( ^18^ F-PSMA) in the pleural hemangioma with a maximum standardized uptake value (SUV _max_ ) of 11.6 (red arrow).

A prostate biopsy was then performed, with an anatomopathological result of prostatic adenocarcinoma, Gleason 9 (4 + 5). The patient underwent a bone scan, which was negative, and an ^18^ F-PSMA PET/CT that showed an area of PSMA hyperexpression in the posterolateral peripheral zone of the middle third of the left lobe of the prostate, with an SUV _max_ (maximum standardized uptake value) of 7.8 and corresponding to the prostatic lesion shown on the MRI. Additionally, a solid thoracic paravertebral nodular formation with PSMA hyperexpression was identified, measuring 15 × 10 mm and with an SUV _max_ of 11.6 ( Figs. 1 , 2 ).

**Fig. 3:**
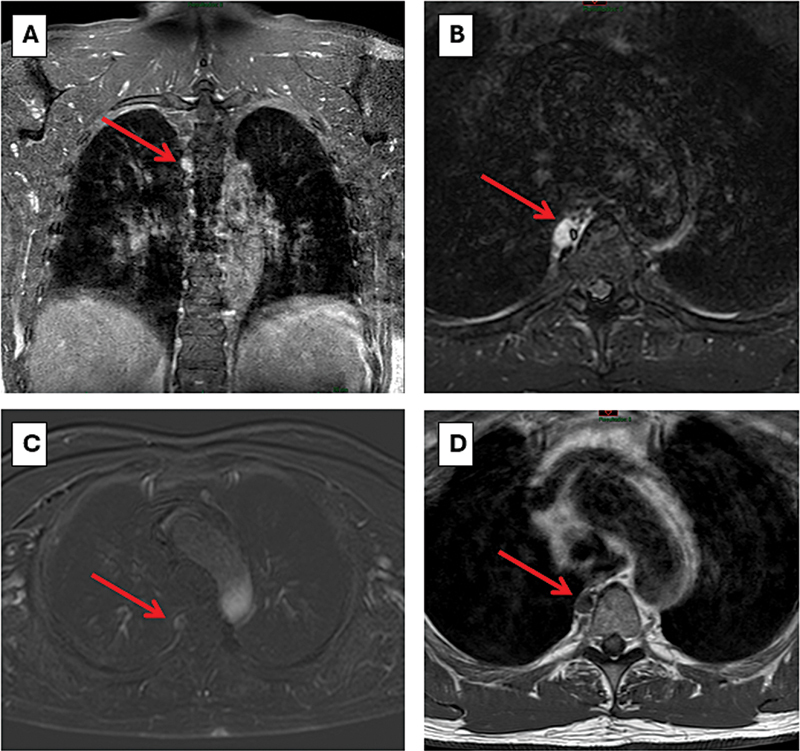
Chest magnetic resonance imaging (MRI) revealed a nodule in the right paravertebral region, at the level of the T4 vertebra (red arrows). It showed contrast enhancement in the portal phase, low signal intensity on T1-weighted images and high signal intensity on T2-weighted images and on the STIR sequence. ( **A** ) Coronal post-contrast T1-weighted image. ( **B** ) Axial STIR sequence. ( **C** ) Axial portal-phase subtraction sequence. ( **D** ) Axial T1-weighted image.

**Fig. 4:**
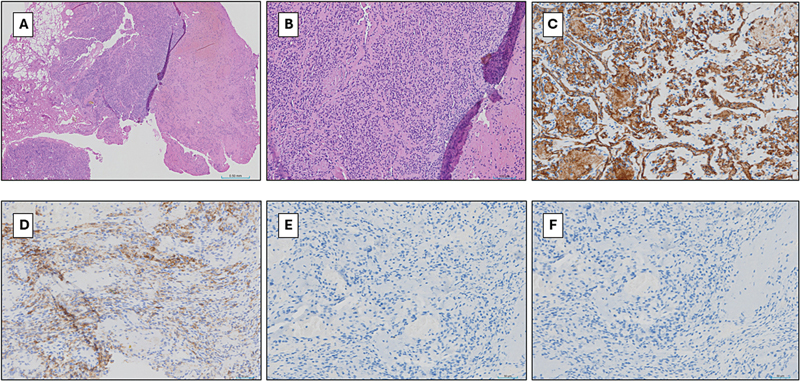
Histological image stained with hematoxylin and eosin from the pleural biopsy, showing an area of chronic pleuritis associated with a vascular proliferation without cytological atypia ( **A** and **B** ). Photographs of immunohistochemical reactions demonstrating positivity for CD31—a vascular marker ( **C** ) and for PSMA ( **D** ). Negativity is observed for the prostatic markers PSA ( **E** ) and NKX3.1 ( **F** ), as well as for pancytokeratin AE1/AE3 (not shown). The final diagnosis was pleural lobular capillary hemangioma.

Due to this finding, the case was brought to a multidisciplinary discussion, leading to the performance of a chest MRI ( Fig. 3 ) and an excisional biopsy of the lesion. The histopathological and immunohistochemical analyses of the lesion confirmed the diagnosis of pleural lobular capillary hemangioma ( Fig. 4 ). The patient then underwent robotic radical prostatectomy, with a first postoperative PSA of 0.06 ng/mL.

## Discussion

Pleural metastases of PCa are uncommon, particularly in the absence of disseminated disease, such as lymph nodes and bones. 9 The detection of a PSMA radiotracer-avid pleural lesion in a high-risk PCa patient immediately raises suspicion of metastatic disease. However, histopathological analysis confirmed it to be a pleural hemangioma. The occurrence of hemangioma in the pleural topography is, by itself, a rarity, with few cases described in literature, generally associated with pleural effusion or identified as incidental findings. To date, PSMA radiopharmaceutical uptake in this specific benign lesion has not been documented, adding pleural hemangioma to the growing list of nonprostatic conditions that can show avidity for this radiotracer.

PSMA radiopharmaceutical uptake in contexts unrelated to PCa is a recognized phenomenon. It is known that PSMA is expressed not only in PCa cells but also in the endothelium of blood vessels formed by neovascularization of different tumors, both malignant and benign, as well as some normal tissues and inflammatory processes. 6 7 As for a pleural hemangioma, the most plausible hypothesis is related to the vascular nature of the tumor. Since it is composed of abnormally proliferated blood vessels, it is reasonable to assume that the endothelial cells of these vessels may express PSMA, resulting in the uptake observed on PET/CT. Associated inflammatory processes or even direct PSMA expression by tumor cells could be alternative mechanisms, but endothelial expression appears to be the most consistent explanation. 10

The clinical implication of the described findings is significant. Misinterpreting a PSMA PET/CT-avid pleural lesion as PCa metastasis could lead to incorrect staging, resulting in erroneous upstaging and leading to inappropriate therapeutic decisions, such as the indication of palliative systemic therapy instead of curative-intent treatment. This case, along with existing reports of pitfalls, reinforces the need for careful correlation of PSMA PET/CT findings with the morphological characteristics of images observed on CT and/or MRI. Furthermore, biopsy should be considered for histopathological confirmation of PSMA PET/CT-avid lesions in atypical locations or with an imaging pattern not characteristic of PCa metastases, even in high-risk patients, aiming for conclusive distinction between prostatic metastasis and a benign pitfall or another malignancy.
